# Human athletic paleobiology; using sport as a model to investigate human evolutionary adaptation

**DOI:** 10.1002/ajpa.23992

**Published:** 2020-01-20

**Authors:** Daniel P. Longman, Jonathan C. K. Wells, Jay T. Stock

**Affiliations:** ^1^ School of Sport, Exercise and Health Sciences Loughborough University Loughborough UK; ^2^ Childhood Nutrition Research Centre UCL Institute of Child Health London UK; ^3^ Department of Anthropology University of Western Ontario London Ontario Canada; ^4^ Department of Archaeology Max Planck Institute for the Science of Human History Jena Germany; ^5^ Department of Archaeology University of Cambridge Cambridge UK

**Keywords:** adaptation, human athletic paleobiology, human evolution, plasticity, sport

## Abstract

The use of sport as a conceptual framework offers unprecedented opportunities to improve our understanding of what the body does, shedding new light on our evolutionary trajectory, our capacity for adaptation, and the underlying biological mechanisms. This approach has gained traction over recent years. To date, sport has facilitated exploration not only of the evolutionary history of our species as a whole, but also of human variation and adaptation at the interindividual and intraindividual levels. At the species level, analysis of lower and upper limb biomechanics and energetics with respect to walking, running and throwing have led to significant advances in the understanding of human adaptations relative to other hominins. From an interindividual perspective, investigation of physical activity patterns and endurance running performance is affording greater understanding of evolved constraints of energy expenditure, thermoregulatory energetics, signaling theory, and morphological variation. Furthermore, ultra‐endurance challenges provoke functional trade‐offs, allowing new ground to be broken in the study of life history trade‐offs and human adaptability. Human athletic paleobiology—the recruitment of athletes as study participants and the use of contemporary sports as a model for studying evolutionary theory—has great potential. Here, we draw from examples in the literature to provide a review of how the use of athletes as a model system is enhancing understanding of human evolutionary adaptation.

## 1 INTRODUCTION

The fossil record provides evidence about the form and physical characteristics of the human or hominin body, and their changes over time. However, a central challenge of hominin paleobiology is the interpretation of the body's function from its form; how do we walk, run, use tools, and move within the landscape, and how did these functions themselves evolve? Stemming from this, what is the nature of the physiological mechanisms that underpin observed variation in form and function?

The use of sport as a conceptual framework offers unprecedented opportunities to improve our understanding of what the body does, shedding new light on our evolutionary trajectory, our capacity for adaptation, and the underlying biological mechanisms.

**Figure 1:**
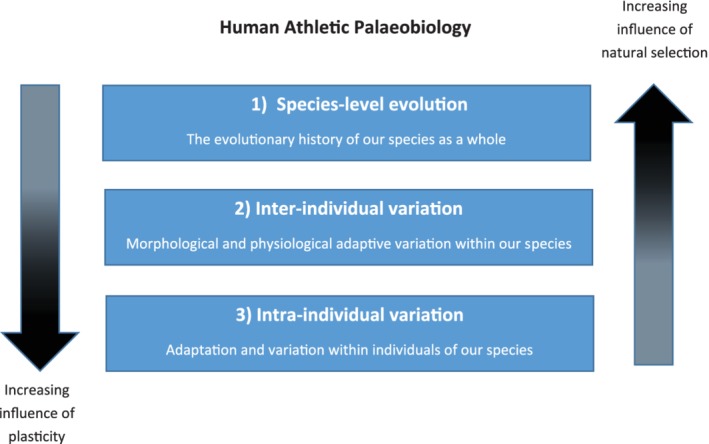
Conceptual diagram highlighting the levels of adaptation studied using sport

There has been increasing interest in the model system provided by athletes to enhance our understanding of human evolutionary theory. To date, studies of athletes have facilitated exploration of three key levels of variation and adaptation within the field of human evolution (Figure 1).

In parallel, different types of adaptation can be investigated through the lens of sports studies. Table 1 highlights this and provides an overview of this article. At the level of species‐level evolution, sports can provide an inferential model for the study of natural selection. At the other end of the spectrum, sports can be used to examine short‐term intraindividual plasticity. It is important to note, however, that the capacity for plastic adaptation also has a heritable component (Wells & Stock, 2011).

**Table 1:** How sports and athletes have been used to study different levels of adaptation

Level of adaptation	How are using sports and athletes being used?	Which component of adaptation is being addressed?	Examples highlighted from the literature
Species‐level evolution	Sportspeople as being representative of past populations	Natural selection acting on lower and upper limbs	Runners and javelin throwers
Interindividual variation	Physical activity as a proxy for subsistence activity in the past	Natural selection, population history, developmental plasticity leading to: Constrained energy expenditure Ecogeographical patterning Intersexual selection	Various endurance competitions
Intraindividual variation	Skeletal analysis of sportspeople to infer adaptations to particular loading patterns, informing interpretation of fossil record	Details how the body adapts through plasticity to demands of subsistence tasks	Cross‐country runners, swimmers, rowers
	Sport as a tool to reveal morphological traits and behaviors that are under selection and the dynamic response to competition	Intrasexual selection	Football, skiing, rowing
	To impose an energetic load and provoke functional trade‐offs	Plasticity	Ultra‐endurance sport, rowing

Firstly, studies utilizing athletic protocols and/or analyzing athletes have elucidated the evolutionary history of our species. This approach has facilitated significant advances in understanding human form and function relative to other hominins in both the upper and lower limbs. Observational and experimental investigations integrating laboratory and field studies with the fossil record have furthered understanding of evolved athletic activities such as bipedal endurance running (Bramble & Lieberman, 2004; Lieberman, 2010, 2012a, 2012b, 2015), the use of projectile technology and spear thrusting (Milks, Parker, & Pope, 2019; Rhodes & Churchill, 2009; Shaw, Hofmann, Petraglia, Stock, & Gottschall, 2012). This methodology has provided new perspectives toward a range of human morphological traits.

Secondly, this approach is shedding new light on human interindividual variation. Variation within the human species may reflect the response to selection acting on different groups in the past (e.g., environmental conditions and sex‐specific energetic pressures). Alternatively, it could also arise through early developmental exposures with effects that may be irreversible later in life (e.g., low birth weight and later‐life constraints in lean body mass), or due to the effects of population history. Investigators in this area have highlighted the influence of physical activity as a selective pressure driving variability in metabolic efficiency (Pontzer, 2015a; Pontzer et al., 2016; Pontzer et al., 2012) and body proportions (Longman et al., 2019).

Thirdly, sport is providing valuable insights into intraindividual variation. Adaptation and variability at the intraindividual level may be more rapid in nature. Much intraindividual variation is achieved via the process of phenotypic plasticity (Hill & Hurtado, 1996). One key adaptive response is the dynamic nature of internal energy distribution in response to environmental energy availability. Life history theory, a branch of evolutionary theory, seeks to characterize the competitive allocation of limited resources between physiological functions throughout the lifespan (Leonard, 2012; Stearns, 1989, 1992; Zera & Harshman, 2001). The study of contemporary sports and athletes in an evolutionary context has the potential to significantly enhance knowledge of our adaptive capabilities as a phenotypically plastic species. This is because athletic events themselves can be used to provoke functional trade‐offs, allowing new ground to be broken in the study of life history trade‐offs and human adaptability.

This article will review the areas of human evolutionary theory already benefitting from the application of this novel approach, considering human variation at the species, interindividual and intraindividual levels in turn. It is hoped that this review will highlight the potential of athletic models not only to complement existing methodologies in human paleobiology, but also to provide unique advances beyond the scope of traditional approaches.

### 1.1 Species‐level evolution

Athletic disciplines, particularly those associated with locomotion and throwing, are providing insights toward the evolutionary history of our species as a whole. This section will review the contributions to current understanding of aspects of the form and function of the human lower and upper limb. In an increasingly sedentary contemporary Western society (Owen, Sparling, Healy, Dunstan, & Matthews, 2010) athletes are often the study participants most aligned with the ancestral populations of interest. The importance of studying athletes with specific training in the activities of interest will be highlighted.

## 2 LOWER LIMB VARIATION

The use of experimental sporting protocols and the analysis of athletes is shedding new light on hominin lower limb morphological variation. Alongside the development of an enlarged and elaborated brain, the adoption of bipedalism is considered a defining characteristic of human evolution (Dart, 1925; Napier, 1967; Rodman & McHenry, 1980a). In his comprehensive review, Niemitz (2010) describes the range of theories that have been suggested to explain this phenomenon. While theories developed before the early 1990s described a savannah environment as the environment of origin of hominin bipedalism (Rose, 1976), it is now generally accepted that a fragmented and variable environment, including woodland, was more likely (Cerling et al., 2010). An explanation which has gained significant traction suggests that hominin bipedalism was instead driven by gains in locomotor efficiency, reducing the cost of foraging (Haile‐Selassie, 2001; Pontzer, Raichlen, & Sockol, 2009; Senut & Pickford, 2004; Sockol, Raichlen, & Pontzer, 2007). Toward the end of the Miocene, when the cooler and drier climate may have made food patches more sparse (Cerling et al., 1997), energetic savings in locomotion would have been increasingly beneficial. This allowed early hominins to travel further distances to find food sources (Rodman & McHenry, 1980b).

This section will draw examples from the literature to highlight how both the deployment of sporting protocols and the analysis of athletes is driving understanding of the development of this key feature of our evolutionary history.

Firstly, anthropological investigations employing techniques typically found in kinesiology and sports science laboratories have shone new light on lower limb evolution. Studies using treadmills and respiratory gas analysis have compiled evidence supporting the theory that lower limb mechanical efficiency may have been driven by selective pressures enhancing locomotion economy when both walking and running (Bramble & Lieberman, 2004; Pontzer, 2007; Steudel‐Numbers & Tilkens, 2004; Steudel‐Numbers, Weaver, & Wall‐Scheffler, 2007; Will, Pablos, & Stock, 2017; Wright & Weyand, 2001). Although it is possible that the shorter lower limbs of *Australopithecus afarensis* conferred an economical advantage when walking (albeit with the disadvantage of a slower walk‐run transition speed; Jungers, 1982; Kramer & Eck, 2000; Kramer, 1999; Ward, 2002), there have been several reports that the longer legs which emerged later in the *Homo* lineage brought demonstrable benefits at a variety of walking speeds (Steudel‐Numbers & Tilkens, 2004). Elongated lower limbs confer an increased optimal walking pace (Bramble & Lieberman, 2004) and enhanced running efficiency across a range of species (Pontzer, 2007; Steudel‐Numbers et al., 2007). While the rationale behind this is complex (Pontzer, 2005), an intuitive explanation is that an individual with longer legs has to take fewer strides to cover a given distance, and would match the speed of a smaller individual while maintaining a lower cadence (see Bramble & Lieberman, 2004; Heglund & Taylor, 1988; Higgins & Ruff, 2011; Jungers, 1982). This results in increased locomotor efficiency as the energetic requirement of a single step is a function of leg length and is largely invariant (Weyand, Smith, Puyau, & Butte, 2010).

Selective pressures acting to improve walking efficiency (Schmitt, 2003; Susman, Stern, & Jungers, 1984), and those favoring the development of endurance running ability (Bramble & Lieberman, 2004), may explain the transition to modern locomotor anatomy from *Australopithecus* to *Homo* (Lieberman, 2015). Consequently, contemporary human habitual bipedalism is distinctive and efficient among extant mammals. Our optimal walking speed is approximately 20% faster and four times more efficient than our closest living relatives, the chimpanzee (Pontzer, Raichlen, & Rodman, 2014; Rubenson et al., 2007; Taylor, Heglund, & Maloiy, 1982). It is noted, however, that Halsey and White (2012) have suggested that the hominin locomotion does not differ in its locomotion costs in comparison with other mammals.

The value of studying athletes has been demonstrated in the barefoot running literature. Humans have been performing endurance running for more than two million years, either barefoot or with minimal footwear over the last 50,000 years (Bramble & Lieberman, 2004; Pinhasi et al., 2010; Trinkaus, 2005). However, the last 50 years has seen the running market flooded with specialist running shoes exhibiting thick cushioned soles and heals (Shawcross, 2014). Dan Lieberman's group has been at the forefront of research investigating the biomechanical consequences of this technological innovation, making significant advances through investigation of trained runners.

Considering first the biomechanical effect of wearing running shoes, Lieberman argues that the cushioning and support creates an environment to which the human foot is not evolved (Lieberman, 2014). Comparative analyses of the biomechanics of running with no or minimal footwear with running shoes have revealed key differences in running techniques. Patterns of barefoot running are typically characterized by a forefoot or mid‐foot strike before the heel contacts the ground. Rearfoot striking, in which the heel contacts the ground first, is rare. In contrast, running in cushioned trainers is associated with an increased tendency to land on the heel. Rearfoot striking generates a rapid and high‐impact ground reaction force that must be absorbed by the skeleton. In contrast, forefront striking, in which the ball of the foot contacts the ground before the heel, avoids the generation of impact peaks as the foot is in a more plantarflexed position upon landing and ankle compliance is increased (see Perl, Daoud, & Lieberman, 2012). Even on hard surfaces, shod rearfoot striking generates larger collision forces than barefoot forefront striking, potentially increasing the risk of injury (Aibast et al., 2017; Lieberman et al., 2010; Lieberman, 2012b; Perl et al., 2012).

While analyzing running foot strike biomechanics, Lieberman and colleagues found that different foot strike angles were observed depending on whether or not the participant was an athlete. Study participants with higher levels of previous running experience, and with faster mile times, were more likely to forefoot or mid‐foot strike than those who were less athletic (Lieberman et al., 2015).

This illustrates an important point—sportsmen and sportswomen are invaluable to anthropologists because they are more representative of past populations than the average person. Numerous metrics indicate that contemporary Western society is growing more sedentary. Jobs are becoming less physically demanding, screen time is increasing (time spent using a computer or phone, watching a television or playing video games), as is time spent sitting (see Owen et al., 2010). As a result, the average member of sedentary Western society may not be a representative model for investigations of biomechanical and energetic aspects of evolved habitual activities such as running, or even walking. The fundamental differences in the running gait between those who do and do not regularly run highlights the value of examining athletes when attempting to model behavior from our past. To gain more accurate insight into the evolved characteristics of locomotion, it is necessary to recruit study participants with repetitive habitual activity profiles more aligned with our active ancestors.

## 3 UPPER LIMB VARIATION

Investigations employing sporting protocols and analyzing athletes trained in the relevant discipline have also provided insights into hominin morphological variation in the upper limb. This new approach is challenging pre‐existing behavioral interpretations of physical traits found in the fossil record. Two examples from the literature will be reviewed to illustrate the value of the model system provided by athletes.

Relative to Holocene *Homo sapiens*, *Homo neanderthalensis* exhibits significant asymmetry in the strength of the humeral diaphysis, as well as asymmetry in anteroposterior strengthening as observed in humeral diaphyseal shape (Churchill & Rhodes, 2009; Churchill, 2002; Churchill, Weaver, & Niewoehner, 1996; Rhodes & Churchill, 2009; Trinkaus, Churchill, & Ruff, 1994). This degree of asymmetry is so pronounced that it is only mirrored in contemporary tennis players and cricketers; groups who regularly load their upper limbs in an asymmetrical manner (Shaw & Stock, 2009a; Trinkaus, 1984).

A proposed explanation for this asymmetry is the use of projectile technology. The use of hand‐thrown spears during the Pleistocene is considered an important event in human evolution (Milks et al., 2019), and could provide an explanation for this observed asymmetry. Prior to the relatively recent invention of the atatl and bow (Shea, 2006), spears facilitated activities such as hunting (Bunn & Gurtov, 2014; Gamble, 1987; Iovita & Sano, 2016; Thieme, 1999), defense against predators (Serangeli, Van Kolfschoten, Starkovich, & Verheijen, 2015), and interpersonal violence (Churchill, Franciscus, McKean‐Peraza, Daniel, & Warren, 2009). However, initial investigations of the effective range of thrown spears, observing that reasonable accuracy was only possible over 5–10 m, discounted throwing as an explanation for observed Neanderthal upper limb asymmetry and provided evidence that spear thrusting could engender similar patterns of asymmetry in mechanical loading (Churchill, 1993).

Recognizing the important role played by skill and training, Milks and colleagues challenged this perception by studying trained male javelin throwers to attempt to hit a hay bale from a series of distances with replicas of the Schöningen Spear II (Schoch, Bigga, Böhner, Richter, & Terberger, 2015; Thieme, 1997). The results revealed that trained throwers were able to throw spears as least twice the distance previously reported and suggest that further improvements would be possible with target‐specific training. As with the analysis of runners to examine evolved barefoot running biomechanics (Lieberman et al., 2015), examination of activity‐specific athletes, more aligned with the activities of our ancestors, provides greater value and insight. It is worth noting, however, that fewer than 25% of attempts from 20 m hit the target, and as Churchill notes, it is unclear how many of these successful hits would have been sufficiently powerful to penetrate an animal's hide (Yong, 2019).

The debate surrounding humeral torsion and retroversion provides a second example from the upper limb literature illustrating the value of studying athletes. Humeral torsion reflects the angular difference between the orientation of the proximal humeral head and the axis of the elbow at the distal humerus (Roach, Lieberman, Gill, Palmer, & Gill, 2012). The degree of humeral torsion has changed during evolution of the *Homo* lineage, with modern humans displaying greater torsion than early *Homo* (Larson, 2007, 2009).

Within *Homo sapiens*, humeral torsion shows great variation, and increases with age between birth and adulthood. Activity patterns throughout life also influence the degree of torsion, likely as a result of a functional imbalance between muscles involved in medial and lateral rotation (Birch, 1997; Cowgill, 2007; L'Episcopo, 1934). For example, tool use such as knapping generates strong internal rotational forces acting to modify the humerus, and increase humeral torsion (discussed in Roach & Richmond, 2015). In contrast, overhand throwing leads to a posterior orientation of the humeral head, generating external rotational forces and decreasing the humeral torsion of the dominant arm by 10°–20°. As a result, individuals regularly performing overhand throwing during adolescence and young adulthood acquire decreased humeral torsion in the throwing arm, leading to high levels of bilateral torsional asymmetry (Bigliani et al., 1997; Borsa, Dover, Wilk, & Reinold, 2006; Borsa et al., 2005; Brown, Niehues, Harrah, Yavorsky, & Hirshman, 1988; Chant, Litchfield, Griffin, & Thain, 2007; Crockett et al., 2002; King, Brelsford, & Tullos, 1969; Magnusson, Gleim, & Nicholas, 1994; Osbahr, Cannon, & Speer, 2002; Pieper, 1998; Reagan et al., 2002). Studies analyzing trained baseball players determined that low humeral torsion enhances elastic energy storage in the shoulder's soft tissues, enabling faster throwing (Roach & Lieberman, 2014; Roach, Venkadesan, Rainbow, & Lieberman, 2013).

Collation of evidence from developmental studies and throwing performance allowed the synthesis of a new argument to counter previous interpretations of the fossil record. Instead of torsional asymmetry being interpreted as a skeletal hallmark of throwing (Rhodes & Churchill, 2009), torsional asymmetry may reflect the opposing effects of

1. The internal rotational forces arising from recent tool use which lead to higher torsion in the nondominant arm, and
2. The external rotational forces stemming from throwing serving to maintain lower torsion in the dominant arm (Roach & Richmond, 2015).

In this way, the model system provided by athletes has provided unique insight and interpretation of function from form in the fossil record, contributing to our understanding of human evolution. The extinction of our *Homo* relatives brings variation within our species into sharper focus, and variation at the interindividual level will be considered in the next section.

## 4 HUMAN INTERINDIVIDUAL VARIATION

Significant morphological and physiological variation exists within the human species (Eveleth & Tanner, 1990; Katzmarzyk & Leonard, 1998; Ruff, 2002; Wells, 2012), and this applies equally to the fossil record of ancestral hominins, even though the evidence is more spare. Such interindividual variation may arise following past selection or following exposure to environmental stresses early in life that irreversibly influence later‐life phenotype. Studies employing sports and physical activities to answer questions related to each of these will now be considered.

## 5 EVOLVED CONSTRAINTS OF ENERGY EXPENDITURE

Hominin evolutionary history is characterized by repeated cycles of dispersal and colonization of new environments (Wells & Stock, 2007). This evolutionary strategy leads to exposure to energetic stress, which may push physiological functions to the limits of adaptive plasticity. Examples of such stressors include unreliable food availability or increased energy demands, and climactic stressors such as extremes of temperature. The significant role played by energy homeostasis in the process of evolution has long been recognized. Building upon the work of early proponents of the idea that energy is critical to the development of species (Boltzmann, 1886; Lodge, 1906), Lotka wrote that “…the fundamental object of contention in the life‐struggle, in the evolution of the organic world, is available energy” (Lotka, 1922).

Strong selective pressures encourage the effective capture and appropriate distribution of energy and resources between competing physiological processes, relating to reproduction, maintenance, growth and defense, though these “allocation decisions” may include the storage of reserves for use at a later date (Leonard, 2012; Leonard & Ulijaszek, 2002; Stearns, 1989; Ulijaszek, 1995; Wells, Nesse, Sear, Johnstone, & Stearns, 2017; Zera & Harshman, 2001). The individual is challenged to develop efficient and effective strategies to acquire and distribute energy and resources toward these key life processes; those that have developed effective systems for both energy acquisition and optimal allocation are thereby advantaged in their particular ecological niche (Angilletta, Wilson, Navas, & James, 2003; Kaplan & Gangestad, 2005; Lotka, 1922).

Traditional models of energy expenditure are additive in nature, considering total energy expenditure as the sum of the energetic demands of an individual's basal metabolism and daily activities (FAO/WHO/UNU, 2001). It would follow that highly active populations expend more energy than groups with comparatively sedentary lifestyles. It was quite striking, therefore, when Pontzer and colleagues found the standardized total daily energy expenditure of Hadza hunter gatherers to be similar to that of Western populations, despite significantly higher estimated physical activity levels (Pontzer et al., 2012).

**Figure 2:**
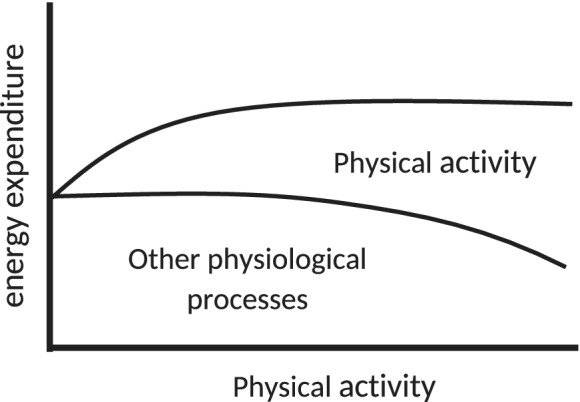
The constrained total energy model, adapted from Pontzer et al. (2016)

Further investigation, considering the energetics of populations with differing levels of habitual physical activity, identified an apparent limit to daily energy expenditure (Pontzer, 2015a, 2015b; Pontzer et al., 2016). The resultant constrained total energy expenditure model proposes that daily energy expenditure is homeostatically maintained within a narrow evolved physiological range. In this model, daily energy expenditure is maintained when levels of physical activity increase, bringing the benefits of reducing energy requirements and pursuant reduced mortality risk (Figure 2).

Consistent with these reports, recent analysis of energy expenditure during pregnancy (Dunsworth, Warrener, Deacon, Ellison, & Pontzer, 2012) and by athletes participating in contests ranging from half‐day triathlons to multimonth ultra‐endurance runs revealed an ultimate limit of sustainable energy expenditure (Thurber et al., 2019). Thurber and colleagues found that over time the maximal daily energy output decreases curvilinearly to a value below three times basal metabolic rate. This level of energy expenditure persists despite ongoing physical activity.

When coupled with high levels of physical activity, this apparent restriction of daily total energy expenditure requires dynamic redistribution of limited available energy within the body. Not all biological functions can continue to receive “normal” levels of energetic funding. Priority is given to biological functions offering the greatest immediate survival value. The constrained total energy expenditure model does not explain this process, and this insight underpins the work of Longman, Stock, and Wells, whose research seeks to determine the hierarchy of functional preservation under conditions of energetic stress (Longman, Prall, et al. 2017), as discussed in more detail later in this review.

## 6 THERMOREGULATORY ENERGETICS, PHYSICAL ACTIVITY, AND SELECTIVE PRESSURES FOR MORPHOLOGICAL THERMAL ADAPTATION

The ability to achieve effective thermoregulation is a key challenge facing individuals in different environments. The physiological processes associated with the maintenance of core body temperature impose a significant energetic burden (Hill, Muhich, & Humphries, 2013). Here, we review studies of physical activity and exercise as proxies for subsistence tasks. As physical activity, influences heat generation and hence the degree of thermoregulatory activation, there is a dynamic relationship between physical activity, thermoregulation and energy expenditure (McArdle, Magel, Gergley, Spina, & Toner, 2017; McArdle, Magel, Spina, Gergley, & Toner, 1984). Depending on ambient conditions and activity levels, thermoregulation has the potential to form a considerable component of human daily energy expenditure, while also constraining the amount of energy that can be directed to other functions.

In cold climates, the heat produced by physical activity contributes significantly to the maintenance of core body temperature, reducing the thermoregulatory energetic load (Tikuisis, Jacobs, Moroz, Vallerand, & Martineau, 2000; Toner, Sawka, Foley, & Pandolf, 1986). In contrast, in warm climates exercise‐induced heat production increases the thermoregulatory burden, and hyperthermia may ensue if the rate of heat production exceeds the rate of heat loss (Montain, Sawka, Cadarette, Quigley, & McKay, 1994).

Ocobock has made important contributions to the field of evolutionary energetics through analysis of the interactions between physical activity, thermoregulatory energy costs, and total daily energy expenditure. Using medical technology designed to monitor patterns of physical activity (namely ActiTrainer devices), Ocobock studied total energy expenditure and its components in highly active people living and working in cold, temperate, and hot environments. The heat produced during exercise was found to be sufficient to differentially influence thermoregulatory costs in hot and cold environments (Ocobock, 2016).

The high energetic costs of active thermoregulation reduce the energy available for other physiological processes. We propose that the selective pressures this generated led to the adoption of temperature‐adapted morphologies in a range of species, acting to reduce thermal stress. Recent investigations of ultra‐endurance running performance in hot and cold environments have provided a unique perspective on the selective forces driving the emergence of these temperature‐adapted morphologies (Longman et al., 2019).

Both extinct and extant hominin species demonstrate morphological traits consistent with Bergmann's (Bergmann, 1847) and Allen's (Allen, 1887) rules (Foster & Collard, 2013; Holliday, 1997a, 1997b; Holliday & Trinkaus, 1991; Tilkens, Wall‐Scheffler, Weaver, & Steudel‐Numbers, 2007). These ecogeographical rules describe patterns of morphological variation with respect to environmental temperature and are based on the principle that heat production is proportional to body mass (heat is produced through cellular activity) and is lost in proportion to body surface area. Consequently, endotherms in warmer climates are proposed to be smaller (Bergmann, 1847) and have longer limbs (Allen, 1887) than those living in colder climates. Early work in humans has demonstrated that annual temperature correlates negatively with body mass (Roberts, 1953) and positively with leg length (Roberts, 1973, 1978). Subsequent research has broadly supported the applicability of Bergmann's and Allen's rules to humans (Crognier, 1981; Foster & Collard, 2013; Hiernaux, 1968; Hiernaux & Fromont, 1976; Katzmarzyk & Leonard, 1998; Ruff, 1994; Stinson, 1990; Trinkaus, 1981).

The mechanisms underpinning morphological adaptation to environmental temperature are unclear and have tended to be relatively adaptationist in nature. For a time, the majority of studies considered natural selection to be the driving force behind ecogeographical patterning (Ashton, Tracy, & Queiroz, 2000), however it is likely that developmental plasticity also plays an important role (Paterson, 1996). Vasomotor changes, functioning to vary the supply of growth factors and blood nutrients, were considered to mediate temperature‐growth effects (Trinkaus, 1981; Weaver & Ingram, 1969). However, more recent experimental work performed by Serrat et al. (2008) suggests that vasoconstriction and vasodilation effect temperature‐mediated changes in growth, not because of variation in the delivery of essential growth‐related blood constituents, but instead by inducing variation in the temperature within developing cartilage (Serrat, King & Lovejoy, 2008).

The majority of studies analyzing the influence of environment on natural selection have to some degree assumed that all groups are consistent in their genetic relatedness and have evolved by natural selection. In reality, population history and structure leads to genetic dependencies between the mean phenotypic values of human groups, which in turn influences patterns of morphological variation (Betti, von Cramon‐Taubadel, & Lycett, 2012; Betti, von Cramon‐Taubadel, Manica, & Lycett, 2013). Through the application of computer simulations and generalized linear mixed models, Roseman and Auerbach (2015) found that population structure explains a significant proportion of among‐group morphological variation. As a result, human ecogeographic patterning cannot be entirely explained by clinally distributed natural selection, but rather is multifactorial and population‐history contingent (Roseman & Auerbach, 2015).

By analyzing the performance of runners competing in multiday ultra‐foot races in hot and cold environments, Longman et al. (2019) recently demonstrated the functional benefits of ecogeographical patterning in thermally challenging environments for the first time. Consistent with the hypothesis that climate‐appropriate body types would reduce thermoregulatory load, morphologies consistent with Bergmann's and Allen's Rules were associated with enhanced performance in hot and cold environments in both sexes (Longman et al., 2020, under review).

**Figure 3:**
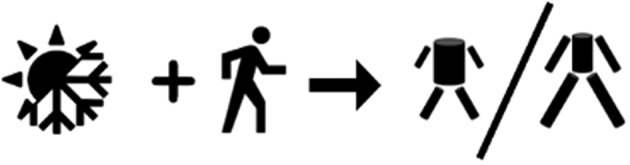
Prolonged physical activity in thermally challenging environments provides the selective pressure for the generation of morphologies through natural selection or developmental plasticity. Taken from Longman et al. (2019)

The physiological demand for heat conservation and dissipation is intensified during prolonged physical activity, generating powerful selective forces that could have driven the emergence of temperature‐adapted morphologies. It may therefore be the interaction between environment and prolonged physical activity that led to the development of environmentally appropriate morphologies (rather than adaptation to an environment allowing for resultant activity). This hypothesis is described in Figure 3.

In addition to providing unique insight regarding morphological adaptation, this study of ultra‐endurance performance also underlined the unique reproductive energetic context of human female adaptation. While both male and female athletes exhibited ecogeographical patterning as predicted by Bergmann's and Allen's rules, the relationship was more pronounced in female athletes; climate‐appropriate morphologies appear to enhance ultra‐running performance to a greater degree in female athletes (Longman et al., 2020, under review). Despite the smaller sample size, the female subgroup displayed a greater number of statistically significant relationships relating to both Bergmann's (weight, BMI, hip circumference, waist circumference, and ponderal index) and Allen's rules (relative leg length). Conversely, the trends in the male data relating to Bergmann's rule did not achieve statistical significance (Longman et al., 2019).

These data suggest that human women have greater sensitivity to thermal stress than men. An explanation for this stems from an adaptive perspective, considering dimorphism in sex‐specific evolutionary trajectories relating to energetic biology. Humans exhibit significant sexual dimorphism in relation to body composition. On average, men have a lower fat mass and a greater lean mass relative to body weight than women, as well as being taller and having increased bone mineral content (Gustafsson & Lindenfors, 2004; Maynard et al., 1998; Rico, Revilla, Hernandez, Villa, & Del Buergo, 1992; Stini, 1972; Wells, 2007). These differences have implications for thermoregulation during physical activity in thermally challenging environments. Sexual dimorphism in body composition and sensitivity to thermal stress may have stemmed from differential selective forces in our evolutionary past, arising from the need for female fat accumulation to buffer infant energy supply from ecological fluctuations (Wells, 2010).

## 7 INTERSEXUAL SELECTION—SIGNALING GENETIC QUALITY

Status within a social hierarchy has important implications for male reproductive success in a range of animal populations (Ellis, 1995; Strier, 2003). The enhanced reproductive success enjoyed by those with high status stem from factors such as increased access to resources, reduced harassment from other group members, and reduced risk of predation. The consequent health benefits lead to an enhanced probability of copulation, conception, and birth of healthy progeny (Ellis, 1995). Similarly, male social status in human groups such as the!Kung of the Kalahari and the Aché of Paraguay is positively associated with the number of surviving offspring, and in contemporary Western societies increasing male income promotes proxies of reproductive success and desirability as a marriage partner (Buss, 1989; Buunk, Dijkstra, Fetchenhauer, & Kenrick, 2002; Hopcroft, 2006; Kaplan & Hill, 1985a; Nettle & Pollet, 2008; R. Pennington & Harpending, 1993; Pollet & Nettle, 2008; Vining, 1986).

Prior to agriculture, hunting may have been a key mechanism for the display of male resourcefulness and the acquisition of social status. Although successful hunters have been shown to enjoy heightened reproductive success (Hill & Kaplan, 1993; Kaplan & Hill, 1985b; Smith, 2004), the mechanism linking the two traits was unknown. On the one hand, the “direct provisioning hypothesis” asserted that successful hunters are more able to share food with their mate and offspring, enhancing reproductive success through physiological means (Hawkes, 1993). Conversely, as successful hunters benefit the community through the sharing of meat in many forager societies (Kaplan & Hill, 1984), hunting success may act as a reliable signal of underlying desirable traits such as athleticism (endurance running may be an important contributor to hunting success [Lieberman & Bramble, 2007]), intelligence or altruism. Based on Zahavi's “handicap principle” (Zahavi, 1975), the elevated social status attained may attract potential mates because of the benefits of association (e.g., protection; Blurton Jones, Marlowe, Hawkes, & O'Connell, 2000).

**Figure 4:**
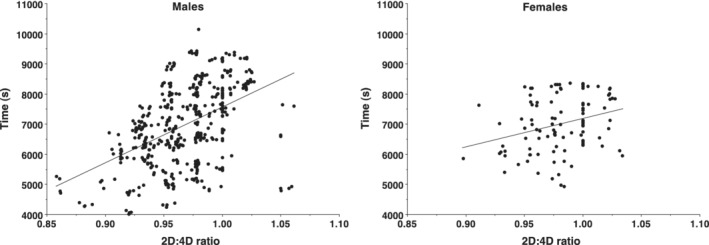
Scatter plot of male and female right hand 2D:4D ratio versus half‐marathon performance (s). The steeper male gradient is visible. Taken from Longman et al. (2015)

A research design studying athletes provided a valuable contribution toward the clarification of this question. Runners competing at a large‐scale half‐marathon were recruited to elucidate the nature of the link between hunting success and elevated reproductive fitness. The large sample size made accessible through this methodology (*n* = 542; *m* = 439, *f* = 103) allowed for a meaningful sex comparison to be made (Longman, Wells, & Stock, 2015; Figure 4).

This study found that a marker of prenatal testosterone exposure (2D:4D ratio) is associated with endurance running ability; an attribute that has been ethnographically shown to be an important component of hunting ability (Liebenberg, 2006). As testosterone has repeatedly been linked with reproductive success (see Manning & Fink, 2008), this investigation provides mechanistic evidence in support of the theory that running capability may serve as a reliable signal of male reproductive quality and potential (Longman, Wells & Stock, 2015). Due to the egalitarian nature of many forager societies, in which meat is widely distributed throughout the group (Kaplan & Hill, 1984), this work suggests that women may be attracted to men with the capacity to acquire resources, rather than those who have resources. It is worth noting that recent work, assessing the link between Hadza hunting reputation, bow pull strength and bow and arrow aiming skill found no association with digit ratio in a relatively small sample (Stibbard‐hawkes, n.d.). A study with a larger sample size is needed to clarify this.

## 8 INTRAINDIVIDUAL VARIATION

The model system provided by athletes and contemporary sporting events is proving increasingly valuable in studying variation at the intraindividual level. Adaptation within an individual's lifetime may be mediated by the process of phenotypic plasticity, whereby an individual's genome produces different phenotypes in response to exposure to varying environmental cues (Pigliucci, Murren, & Schlichting, 2006). Plasticity can be mediated at the behavioral, biochemical, physiological, or developmental levels, each differing in the degree of reversibility (Pigliucci, Murren & Schlichting, 2006). Commonplace human examples of phenotypic plasticity include immune system adaptation to pathogen exposure, as well as mechanisms of learning (Fusco & Minelli, 2010). Although not all plasticity is beneficial (Sultan, 1995), the ability of an individual to modify its phenotypic status in response to changes in the environment affords great adaptive potential and responsiveness to nonstatic conditions (Garland & Kelly, 2006). In this section we discuss recent advances provided by athlete‐based models of investigation concerning the plasticity of long bone structure, intrasexual selection and life history theory. It is important to note that plasticity influences variation at both the interindividual and intra‐individual levels, and that there is no intention to imply a hard‐dividing line between these two sections.

## 9 SPORT AS A TOOL TO INFER BEHAVIORAL PATTERNS FROM THE FOSSIL RECORD

Biomechanical movement creates strain in bones, both through muscle contraction and ground reaction forces, and these have been measured in vivo using implanted strain gauges (Burr et al., 1996; Lanyon, Hampson, Goodship, & Shah, 1975; Rubin & Lanyon, 1984). Mechanical loading is consistently linked with cross‐sectional limb bone size, rigidity, and shape (Hseih, Robling, Abmbrosius, Burr, & Turner, 2001; Jones, Priest, Hayes, Tichenor, & Nagel, 1977; Judex, Gross, & Zernicke, 1997; Nikander, Sievänen, Uusi‐Rasi, Heinonen, & Kannus, 2006; Shaw & Stock, 2009a, 2009b; Woo et al., 1981). Considerable experimental evidence demonstrates that long bone diaphyses display plasticity within a lifetime and adaptively respond to increased loading by structurally augmenting their mass in the direction of the deformation (Lanyon, 1992; Rubin, McLeod, & Basin, 1990). The organization of bone tissue may be driven by, and be reflective of, the range of daily strains imposed by a wide range of activities imposing different strain intensities (McLeod, Rubin, Otter, & Qin, 1998).

Studies seeking to infer patterns of activity in prehistoric hominin groups depend upon comparative analysis of skeletal morphology (Churchill et al., 1996; Holt, 2003; Holt & Formicola, 2008; Marchi, 2008; Ruff, 2008, 2009; Trinkaus, Churchill & Ruff, 1994). The interpretive power of such studies has been enhanced by comparison of the morphology evolutionary and archaeological hominin record to that of living groups (see Ruff, 2000; Ruff, Trinkaus, & Holliday, 1998; Trinkaus et al., 1994), whose activity patterns and skeletal morphology are well documented (Shaw, 2010, 2011; Shaw & Stock, 2009a, 2009b).

The study of athletes, whose discipline‐specific training has subjected their long bones to a particular set of forces, is providing valuable comparative data from which to infer prehistoric activity patterns. This approach is exemplified by work performing a comparative analysis of interlimb patterns of robusticity (tibial J/humeral J) in living athletes (cross‐country runners and swimmers), living controls, Pleistocene fossil hominins (Neanderthals and anatomically modern and Upper Paleolithic *H*. *sapiens*), and Holocene foragers (terrestrial LSA southern Africans and marine Andaman islanders; Shaw & Stock, 2013). As expected, among the living cohort, runners had higher levels of tibial rigidity relative to humeral rigidity than the swimmers. Similarly, the relative tibial rigidity of terrestrial and marine Holocene foragers resembled the athlete groups with comparable patterns of habitual activity (runners and swimmers, respectively). The Pleistocene fossil hominins resembled terrestrial Holocene foragers and runner subsamples. Furthermore, almost half of the Pleistocene individuals sampled displayed tibial rigidities suggestive of volumes of walking/running exceeding that of contemporary runners (80–100 miles per week).

The value of studying analyzing athletes to enhance the interpretation of prehistoric skeletal robusticity has been further demonstrated in work investigating sexual dimorphism and labor across the transition to agriculture (Macintosh, Pinhasi, & Stock, 2017). Comparative analyses with contemporary athletes demonstrated that for over 5,000 years of prehistory in central Europe, women had stronger humeral cross‐sectional properties than contemporary female rowers. The results highlight sex differences in the norms of reaction of bone to patterns of mechanical loading, emphasizing the need for sex‐specific analyses to infer past female behavior from the mechanobiology of skeletal tissue.

Present interpretations of behavioral patterns from fossils often lack consistency (Shaw & Stock, 2009b). In order to further understanding of prehistoric activity patterns, further clarification of the complex relationship between habitual loading patterns and diaphyseal adaptation is required. Competitive athletes repeatedly perform the same activities in training and in competition, often from a young age. As a result, investigations of living athletes can provide detailed information linking habitual activities to diaphyseal morphology. The use of athletes in this way, across a range of disciplines encompassing varying intensities, repetitiveness, and planes of movement, are adding to previous understanding of the influence of habitual activity on diaphyseal rigidity and shape patterns (Shaw & Stock, 2009a). Research in this area has demonstrated that differences in the manner of loading, as well as frequency, are linked to variation in long bone shape (Richmond & Jungers, 2008; Ruff, 1995; Ruff et al., 2006; Trinkaus, 1997). A recent study of trabecular bone microarchitecture in living human distance runners has demonstrated that runners with a forefoot strike, interpreted to have greatest summative loading stimulus due to training, have greater trabecular thickness (Best, Holt, Troy, & Hamill, 2017). Ultimately, this line of research will contribute to a unifying theory explaining the influence of different activities on the skeleton and allow the inference of mobility patterns from hominin skeletal remains.

## 10 SPORT AS A MODEL OF INTRASEXUAL SELECTION

Manning and colleagues have drawn parallels between athletic competition and intrasexual selection, highlighting the similarities between the traits required for success in each. Taking football (soccer) or rugby as examples, to be successful a player must have spatial judgment to pass to and receive the ball from team‐mates, and cardiovascular development and efficiency to play competitively for 90 or 80 min, respectively. Speed to reach the ball first and use it effectively, and strength to shield it from opponents, are also required (Manning & Taylor, 2001). Sport further mirrors abilities in male–male competition through the prominence of actions such as throwing, punching, kicking, and running (Hönekopp, Manning, & Müller, 2006). As all these attributes are beneficial in male–male combat, performance in sport may reflect potential ability in this domain.

Furthermore, intense rivalry often exists between sporting opponents. An official with total control is almost always required to police contests, as competitors constantly test the behavioral limits as dictated by the rules of the contest (Hönekopp, Bartholdt, Beier & Liebert, 2006; Manning & Taylor, 2001). Perhaps unsurprisingly then, athletic ability across a range of sports has been linked to 2D:4D digit ratio (Longman, Stock & Wells, 2011; Manning 2002; Manning, Morris & Caswell, 2007; Manning & Taylor, 2001; Paul, Kato, Hunkin, Vivekanandan & Spector, 2006; Pokrywka, Rachon, Suchecka‐Rachon & Bitel, 2005)—an early life indicator of subsequent reproductive fitness (Berenbaum et al., 2009; Hönekopp et al., 2007; Manning, Scutt, Wilson & Lewis‐Jones, 1998; Manning, Barley, Walton, Lewis‐Jones & Trivers, 2000; Manning & Fink, 2008). Sport also mirrors intrasexual selection insofar as the status‐enhancing and monetary rewards facilitate resource acquisition, promoting access to mating opportunities (Buss, 1989; Edwards, 2006; Manning & Taylor, 2001).

Androgenization, positively linked with reproductive success in many animal populations, is also positively related to status within a social hierarchy (Ellis, 1995; Strier, 2003). Status is often determined by male–male competition (Altmann, Sapolski, & Licht, 1995), and testosterone levels have been shown to closely track the results of such dominance interactions across a range of mammalian species (Zilioli & Watson, 2012).

The validity of sport as a proxy for male–male competition in a selective context is supported by reports of testosterone tracking the outcomes of both athletic and nonathletic contests in contemporary Western societies, as well as in the vicarious experience of winning among sports fans (Apicella et al., 2008; Archer, 2006; Bernhardt & Dabbs, 1997; Bernhardt, Dabbs, Fielden, & Lutter, 1998; Booth, Shelley, Mazur, Tharp, & Kittik, 1989; Elias, 1981; Gladue, Boechler, & McCaul, 1989; Longman, Surbey, Stock, & Wells, 2018; Mazur, Booth, & Dabbs, 1992; Mazur & Lamb, 1980; McCaul, Gladue, & Joppa, 1992). This mirrors the increases in testosterone that have been observed in primates following a dominance interaction (Muller & Wrangham, 2001), and in pre‐industrialized communities following hunting success (Trumble, Smith, Connor, Kaplan, & Gurven, 2013). It is worth noting, however, that nonsignificant differences in testosterone levels between winners and losers have been reported in sporting and video gaming contests (Gonzalez‐Bono, Salvador, Ricarte, Serrano, & Arnedo, 2000; Mazur, Susman, & Edelbrock, 1997; Salvador, Simón, Suay, & Llorens, 1987; Salvador, Suay, & Cantón, 1990; Suay et al., 1999).

Recent work employing a rowing contest as a model of intrasexual selection has enhanced understanding of the dynamic relationship between testosterone, status and a key trade‐off relating to reproductive strategy (the allocation of energetic resources toward either mating or parenting [McGlothlin, Jawor, & Ketterson, 2007]). Perceived victory in an experimentally manipulated head‐to‐head rowing machine competition between young adult male trained rowers led to both a surge in androgenization, as well as psychological changes pertaining to reproductive strategy. Self‐perceived mate value, self‐esteem, inclination toward engaging in casual sexual relationships and increased intention to instigate such relationships all increased in winners, while the propensity toward caring for or mentoring children decreased (Longman, Surbey, Stock & Wells, 2018). The tandem hormonal and psychological shifts in male reproductive effort following victory represent a significant shift to the mating end of the mating‐parenting trade‐off. The use of sport as a model of intrasexual competition not only allowed for analysis of the trade‐off between mating and parenting effort, but also facilitated an experimental design in which the physical effort of winning was uncoupled from the social perception of winning. The utilization of a manipulated competition result highlights that the social experience of winning causes the testosterone surge of a “victory,” and strongly influences reproductive investment and strategy.

## 11 ULTRA‐ENDURANCE SPORT AS A MODEL TO STUDY HUMAN LIFE HISTORY THEORY

### 11.1 Trade‐offs in energy allocation

Life history theory describes the competitive allocation of limited resources between physiological functions (Leonard, 2012; Stearns, 1989, 1992; Zera & Harshman, 2001). During periods of energetic stress, life history theory hypothesizes that trade‐offs between competing processes arise (Bronson, 1991; Stearns, 1992); a life history strategy involving a greater allocation of resources toward a given function necessitates a reduction in the resources available for other functions. Hence, there is a strong selective pressure for energetic efficiency. Limited resources are predicted to be preferentially allocated to biological functions offering the greatest immediate survival value. However, the hierarchy of functional preservation, and how this varies with population, age, sex, and body composition, is unknown.

The energetic cost of reproduction is central to many life history trade‐offs (Stearns, 1989). Although reproduction is only one of the key functions described by life history theory, the other processes are only of value from a fitness perspective in that combined, these processes increase the opportunities for reproduction in the future. Evolutionary theory argues that individuals should, at every reproductive opportunity, exhibit behavior intended to enhance genetic contributions to subsequent generations. However, a life history strategy involving a greater allocation of energetic resources toward reproduction imposes reduced allocation to other functions, such as survival.

While the concept of a life history trade‐offs is appealing, negative correlations between investment in two competing physiological functions are frequently absent when phenotypic comparisons are made between individuals within a population (Cody, 1966; Glazier, 1999). This may be due, at least in part, to the finding that interindividual variation in resource acquisition often exceeds variation in resource allocation (van Noordwijk & de Jong, 1986).

Recently published research (Longman et al., 2017) identified ultramarathon competitions as a valuable experimental model enabling observation of negative covariations between investment in competing physiological functions in the field. Although it is not possible to control individual energy intake without compromising ecological validity (or, perhaps, reasonable research ethics), it is possible to experimentally control energy balance. This innovative model utilizes the pre‐existing energy deficit inherent in ultramarathons (Knechtle & Bircher, 2005; Knechtle, Enggist, & Jehle, 2005) to nullify the effect of variation in resource acquisition. This negative energy balance pushes physiological and cognitive systems to the limits of adaptive plasticity, provoking detectable functional trade‐offs. This allows us, for the first time, to directly test physiological trade‐offs and observe how the body prioritizes different tissues or functions.

**Figure 5:**
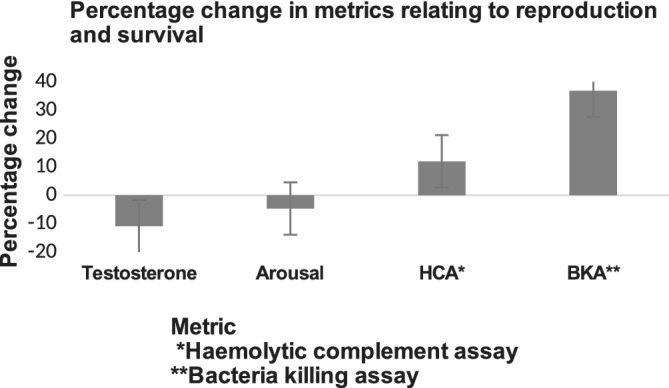
Changes in investment in measures of reproduction (testosterone and arousal) and survival (hemolytic complement assay and bacteria killing assay) following participation in a 100‐mile footrace. Taken from Longman et al. (2017)

We used ultra‐endurance events to study human life history trade‐offs during a 100‐mile foot race. This study revealed an acute‐level trade‐off between reproduction and survivorship in male athletes during which athletes lost body weight. The data highlighted a shift in energetic priorities away from reproduction (as measured by levels of testosterone and libido), and toward short‐term survival (as measured by innate immune function, a marker of defense; Longman et al., 2017). The changes in each of the four metrics achieved statistical significance (Figure 5).

The sample populations tested in ultramarathons are, by their very nature, highly trained and physically fit individuals. The precise results may not therefore be generalizable to the wider population. For example, the high levels of physical conditioning may buffer the study cohort from detrimental health consequences of malnutrition during prolonged and strenuous physical exercise. However, given the universality of general life history theory predictions, one can argue that results in different populations should be broadly comparable due to the evolutionary (Carrier, 1984; Lieberman, Bramble, Rachlen & Shea, 2006; Longman et al., 2015) and cross‐cultural ecological relevance of endurance running (Liebenberg, 2006; Pennington, 1963).

We have since developed this model through the incorporation of more detailed measures of immune function, which promises to shed further light on the survival versus reproductive trade‐off. This work spans a range of environmental conditions, incorporating both multiday ultramarathons and ocean rows spanning more than 4 weeks in duration. In addition, we have actively addressed the need to increase the number of female study participants. Female participation in ultramarathons is often significantly lower than males (Knechtle, Knechtle, & Lepers, 2011). It is worth noting that the model of using ultra‐endurance events to study life history trade‐offs and human adaptability offers a smooth route to navigate the ethical concerns inherent in studying participants under physical stress. This is because the sporting events under investigation are often taking place independently of the study protocol.

As previously described, Pontzer's constrained total energy expenditure model (Pontzer et al., 2016) suggests that daily energy expenditure is maintained within a narrow evolved physiological range. Pontzer suggests that the additional energy demand of increased levels of physical activity is absorbed through metabolic adaptations to save energy in other physiological systems, which are yet to be described (Pontzer et al., 2015). The novel approach to studying life history trade‐offs using ultramarathons is beginning to answer questions concerning this metabolic adaptation. Over the next few years we hope to be able to characterize the hierarchy of functional preservation under conditions of energetic stress. In doing so, this will shed new light on our adaptive capabilities as a phenotypically plastic species.

Ultra‐endurance events have demonstrated that the energy load generated by sporting activities can be used to reveal coherent energetic allocations across *functions*. In parallel, recent experimental work has highlighted that this energy load can also be used to reveal moment‐by‐moment differences in fuel allocation between *tissues*.

The development of an enlarged and elaborated brain is a defining characteristic of human evolution (Foley & Lee, 1991; Hawks, Hunley, Lee, & Wolpoff, 2000; Lee & Wolpoff, 2003; Ruff, Trinkaus, & Holliday, 1997). This has brought a plethora of benefits to the *Homo* clade (Barrickman, Bastian, Isler, & van Schaik, 2008; Byrne & Corp, 2004; Gibson, 1986; Parker & McKinney, 1999; Reader & Laland, 2002), but at the cost of the brain having the highest metabolic requirements relative to size of all organs (Attwell & Laughlin, 2001; Bullmore & Sporns, 2012; Isler & van Schaik, 2006; Mink, Blumenschine, & Adams, 1981). As a result, the issue of how an enlarged and elaborated brain can be metabolically afforded is a prominent and persistent question within human evolution (Aiello & Dunbar, 1993; Aiello & Wheeler, 1995; Byrne, 1997; Isler & van Schaik, 2006, 2011; McNab & Eisenberg, 1989; Navarrete, van Schaik, & Isler, 2011). Skeletal muscle mass is also an expensive tissue to maintain (Elias, 1992; Snodgrass, Leonard, & Robertson, 1999), and, like the brain, its glucose demands increase with activation (Bélanger, Allaman, & Magistretti, 2011; Brooks & Mercier, 1994; McArdle, Katch, & Katch, 2001; Romijn, Gastaldelli, Horowitz, Endert, & Wolfe, 1993; Wahren, Felig, Ahlborg, & Jorfeldt, 1971). During such circumstances, muscle tissue may compete with the brain for glucose and oxygen.

An experimental design applied to a sporting contest was employed to investigate the hypothesis of a trade‐off involving the brain at the acute, rather than the evolutionary or developmental, level. Simultaneous challenge of both cognitive and physical functions resulted in relative preservation of cognitive function over physical power output (Longman, Stock, & Wells, 2017), lending support to the selfish brain hypothesis (Peters et al., 2004) and highlighting the metabolically privileged niche occupied by the human brain. This metabolic hierarchy may be an evolved trait, as the chances of survival may be boosted more by a well‐fuelled brain than well‐fuelled muscles when facing an environmental challenge (Beedie & Lane, 2012).

Research seeking to understand the competitive allocation of resources between key functions is central to life history theory, and cuts to the heart of our nature as a phenotypically plastic, colonizing species (Wells & Stock, 2007). The study of modern sports in this context has the promise to enhance understanding of this process, and consequently knowledge of our plasticity and adaptive capabilities.

## 12 SUMMARY AND FUTURE PERSPECTIVES

A plethora of scientific disciplines have contributed to the study of human evolution, ranging from primatology and bioarcheology to paleontology and genetics. Each approach has added a unique perspective, building knowledge of the origins and development of our species. Here, we have reviewed a new methodology. Human athletic paleobiology—the analysis of athletes as study participants and the use of contemporary sports as a model for studying evolutionary theory—has great potential.

The appeal of utilizing athletes and sport to study human variation is multifactorial. The varying characteristics of the wide range of existing sporting contests offer diverse and unique opportunities as a methodological tool. From a research design perspective, this model facilitates a variety of data collection protocols. These range from field‐based observational studies to laboratory‐based rigorous randomized controlled trials with experimental designs. The opportunity to design and perform controlled experimental investigations is particularly valuable to anthropology. Furthermore, interdisciplinary collaborations are possible through collaborations between researchers in anthropology and sport science, kinesiology, physiology and psychology. This allows for the application of specialized equipment and complementary expertise with the potential to provide alternative, valuable perspectives to our discipline.

From a biological perspective, the range of contemporary athletic events allows for the functional assessment of a variety of different biological systems. For example, it has been proposed that the contrasting morphologies of humans and Neanderthals may reflect the selective pressures imposed by endurance versus sprinting or other power‐related hunting styles (Bramble & Lieberman, 2004; Liebenberg, 2006; Stewart et al., 2018). Insights toward the selective pressures imposed by these opposing hunting styles are made possible by studying athletic physiologies associated with enhanced performance in endurance events (e.g., marathon running) in comparison to power‐based disciplines (e.g., weightlifting, sprinting or rugby). This would allow analysis of the cardiovascular and aerobic system, and of muscular and anaerobic energy systems. In tandem with the large sample sizes available across a range of athletic disciplines, the increased depth and breadth of viable metrics arising from the use of living subjects often allows for the derivation of clearer insights.

To date, this model has been shown capable of enhancing understanding of variation at the species, inter‐individual and intra‐individual levels. The potential to develop this avenue of research is vast. One approach to realize this potential is to establish collaborations with specialized sports science or kinesiology departments. This will allow laboratory‐based investigations to be performed, with the aim of standardizing field measurements and analyzing interesting field observations in greater depth.

Aspects of the literature cited in this article have been selected to illustrate not only how this approach can enhance understanding of our evolutionary past, but also how this knowledge can be forward‐facing in its application. Energetic stress presents as a prominent problem in contemporary society. Situations such as famine, war and migration bring inherent food insecurity, and the potential for energy deficit (Abubakar et al., 2018; The World Bank, 2018). The recent UCL–*Lancet* Commission on Migration and Health stressed that migration and health are inextricably linked, and are key to sustainable development (Abubakar et al., 2018). A comprehensive understanding of biological adaptation to energetic stress, as described at the level of intraindividual variation here, is therefore critical to health and medical outcomes. Evolutionary scientists understand that energy allocation underpins multiple functional relationships, but this perspective has yet to emerge in biomedical science. Increased knowledge of the scope of human plasticity and the adaptive stress response outside the context of overt disease can be applied to numerous areas of public health, enhancing understanding of the interrelationship between body weight regulation, physical activity, dietary intake, and health. This understanding will contribute to the emerging field of evolutionary public health, which is using knowledge derived from life history theory trade‐offs to improve the efficacy of public health interventions (Wells, Nesse, Sear, Johnstone & Stearns, 2017).

## Data Availability

Data sharing is not applicable to this article as no new data was created or analyzed in this study.
